# Embo: a Python package for empirical data analysis using the Information Bottleneck

**DOI:** 10.5334/jors.322

**Published:** 2021-05-31

**Authors:** Eugenio Piasini, Alexandre L. S. Filipowicz, Jonathan Levine, Joshua I Gold

**Affiliations:** 1. Computational Neuroscience Initiative and Department of Physics and Astronomy, University of Pennsylvania.; 2. Toyota Research Institute.; 3. Department of Neuroscience, University of Pennsylvania.; 4. Department of Neuroscience, University of Pennsylvania.

**Keywords:** Information theory, Python, Information Bottleneck, Deterministic Information Bottleneck, data analysis, statistics

## Abstract

We present *embo*, a Python package to analyze empirical data using the Information Bottleneck (IB) method and its variants, such as the Deterministic Information Bottleneck (DIB). Given two random variables *X* and *Y*, the IB finds the stochastic mapping *M* of *X* that encodes the most information about *Y*, subject to a constraint on the information that *M* is allowed to retain about *X*. Despite the popularity of the IB, an accessible implementation of the reference algorithm oriented towards ease of use on empirical data was missing. Embo is optimized for the common case of discrete, low-dimensional data. Embo is fast, provides a standard data-processing pipeline, offers a parallel implementation of key computational steps, and includes reasonable defaults for the method parameters. Embo is broadly applicable to different problem domains, as it can be employed with any dataset consisting in joint observations of two discrete variables. It is available from the Python Package Index (PyPI), Zenodo and GitLab.

## (1) Overview

### Introduction

#### The Information Bottleneck Method

In the Information Bottleneck (IB) framework [1], given two random variables *X* and *Y*, we are interested in extracting all the information that *X* may contain about *Y* and discarding the rest as irrelevant. To solve this problem, we seek a third random variable *M* that solves the following optimization problem:

(1) $$\underset{p(m|x)}{\text{min}}I(M:X)-\beta I(M:Y)$$

where *I*(· : ·) is Shannon’s mutual information [2], and *M* is constrained to be independent of *Y* conditional on *X*:

(2) p(x,m,y)=p(x)p(m∣x)p(y∣x)

**Figure 1:**
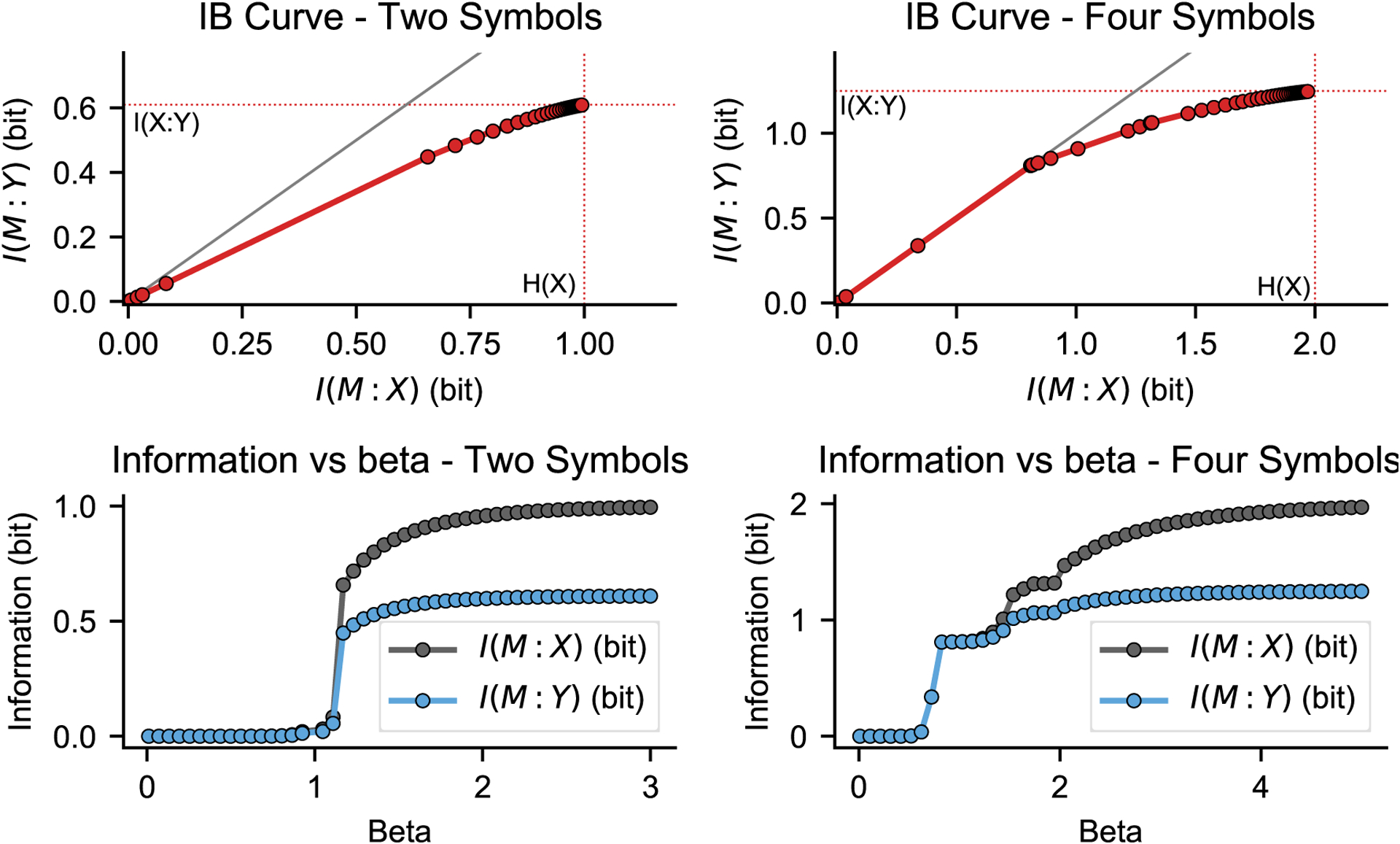
From embo’s documentation (examples/Basic-example.ipynb): Top, red: IB curves for two simple synthetic datasets, one where both *X* and *Y* are binary (left column, “Two symbols”) and one where they can both take on 4 possible states (right column, “Four symbols”). Each dot represents the solution of Equation (1) for a particular value of *β* (solid lines connecting the dors are added for legibility). Gray: identity line. Bottom: values of *I*(*M* : *Y*) and *I*(*M* : *X*) versus their corresponding values of *β*. See the software documentation for further detail on how these figures were generated. Note that the IB curve is always below the identity line and that the values of *I*(*M* : *Y*) and *I*(*M* : *X*) are never larger than the base 2 logarithm of the number of states (1 bit and 2 bit, respectively, corresponding to 2 and 4 states, respectively). These are conditions that the IB curve should always satisfy [1] and can be taken as sanity checks for embo’s correct operation.

Intuitively, Equation (1) says that we are looking for a stochastic mapping of *X* to *M* that keeps as little information about *X* as possible while maximizing the information about *Y*. *β* is an arbitrary (positive) parameter quantifying the relative importance of these two competing goals. In the spirit of rate distortion theory [2], it can be shown [1] that the set of solutions to this method for all possible values of *β* gives an upper bound to the amount of information one can encode about *Y* given a certain amount of information about *X*, or vice versa, the minimum amount of information about *X* needed to encode a certain amount of information about *Y*. These bounds are typically summarized by plotting a curve showing *I*(*M* : *Y*) versus *I*(*M* : *X*), obtained by computing these quantities for the solution of Equation (1) across many different values of *β*. This is known as the *IB curve*. Example IB curves, taken from one of the notebooks in embo’s documentation, are shown in Figure 1.

Because of its appealing theoretical properties, since it inception the IB has enjoyed continued attention as a method for unsupervised [3] and supervised [4, 5] learning, as well as becoming more recently a popular tool in the study of learning and generalization in deep neural networks [6, 7] and in neuroscience [8, 9, 10, 11].

#### Generalized and Deterministic Information Bottleneck

A useful generalization of the Information Bottleneck was introduced by [12]. By noting that *I*(*M* : *X*) = *H*(*M*)−*H*(*M*|*X*), one observes that there are two different ways in which the bottleneck variable *M* can have limited information about *X*: it can have limited variability (small *H*(*M*)), or it can be very noisy (large *H*(*M*|*X*)). These possibilities suggests that we could modify the cost function in (1) as follows:

(3) $$\underset{p(m|x)}{\text{min}}H(M)-\alpha H(M|X)-\beta I(M:Y)$$

where *α* ≥ 0. We call this the Generalized Information Bottleneck problem, or GIB (note that the same acronym is used in [13] with a different meaning). The GIB reduces to the standard IB as a special case for *α* = 1.

**Figure 2:**
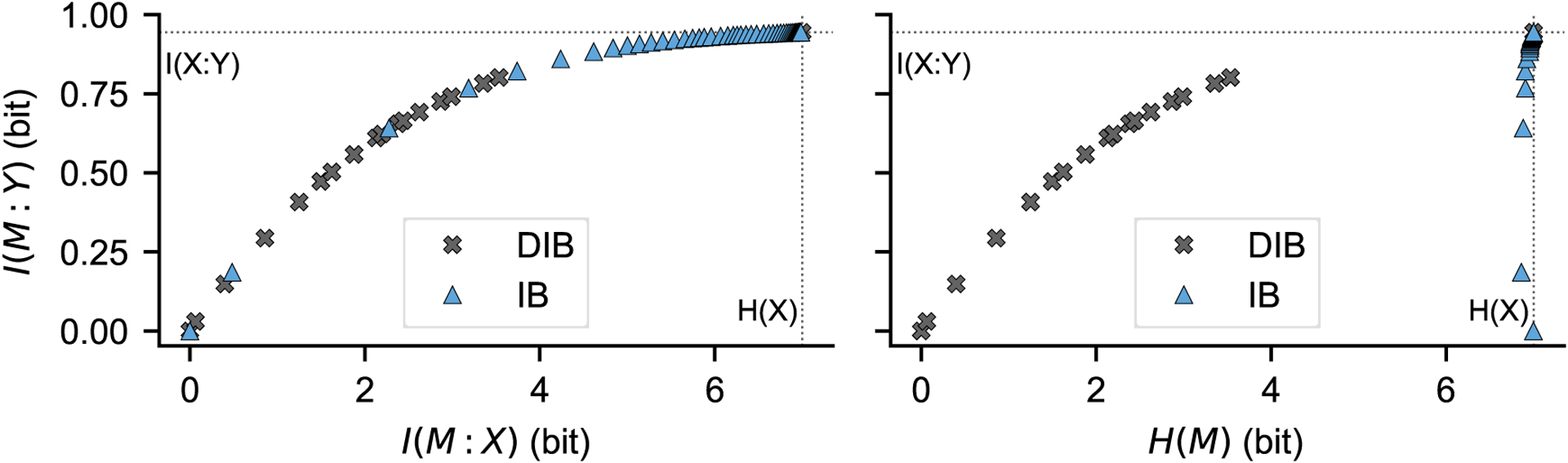
From the documentation (examples/Deterministic-Bottleneck.ipynb): comparison of IB and DIB, similarly to Figure 2 in [12]. In this example, *X* can take on one out of 128 possible states, *Y* can take on one out of 32 states, and *p*(*x*) is close to uniform (see the notebook for details about the joint *p*(*x, y*)). Left: IB and DIB solutions for a range of *β* values, visualized in the “IB plane” where *I*(*M* : *Y*) is plotted against *I*(*M* : *X*). Right: same solutions as in the left panel, visualized in the “DIB plane” where *I*(*M* : *Y*) is plotted against *H*(*M*). As expected from [12], in the IB plane the two methods behave similarly. In the DIB plane, however, the DIB performs better than the IB in the sense that *H*(*M*) is much lower for the DIB than for the IB, for any given value of *I*(*M* : *Y*).

If *α* = 0, the problem consists of finding the minimum-entropy bottleneck variable *M* that contains a certain amount of information about *Y* (or the *M* with the largest amount of information about *Y* among all *M*s with a set entropy). This is called the Deterministic Information Bottleneck (DIB) by [12]. The term “deterministic” comes from the fact that solutions in the *α* = 0 case are shown to be deterministic mappings from *X* to *M*, with *H*(*M*|*X*) = 0. A simple demonstration of application of the DIB, inspired by one of the examples given in [12], is illustrated in Figure 2.

#### IB for empirical data; comparison with other software

**Figure 3:**
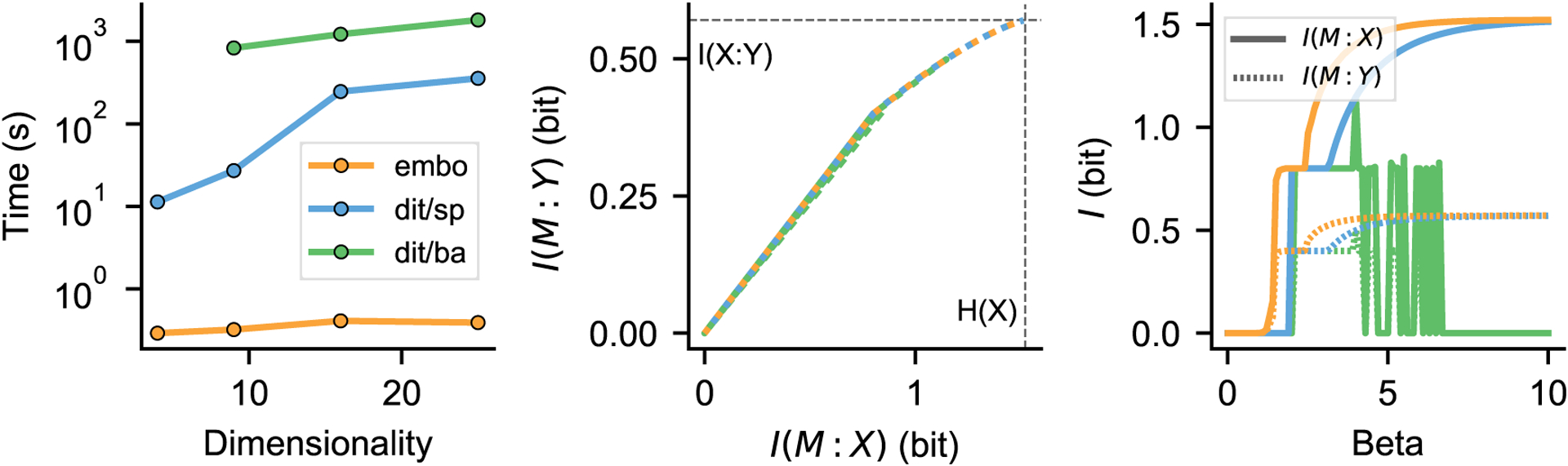
From embo’s documentation (examples/Compare-embo-dit.ipynb): comparison of embo and dit [14] on sample IB problems of different dimensionality, defined as the number of possible states for the joint random variable (*X, Y*). The problem with dimensionality 9 (where both *X* and *Y* have three possible states) is taken from the documentation of the current version of dit. Left: runtime vs dimensionality. Dit/sp and dit/ba indicate the algorithm used by dit: sp for scipy.optimize and ba for the Blahut-Arimoto algorithm. It was not possible to run dit on the smallest problem due to a software bug. Center: IB bound for the problem with dimensionality 9, computed with embo and dit. Embo and dit/sp (blue and orange) find the same solution, while dit/ba (green) finds a suboptimal one. Right: *I*(*M* : *X*) and *I*(*M* : *Y*) as a function of *β*. Note how dit/ba (green) becomes unstable at large *β*. See notebook for more details.

Despite the large body of existing work on the IB (and GIB), public, off-the-shelf implementations of its “reference” version based on the Blahut-Arimoto algorithm [1, 12] have been lacking. The supplementary Python code associated to [12] implements the GIB, but it is rather tightly coupled to the specifics of that paper and is not distributed as a standard package (it does not contain tests or licensing information, and is not available on the Python Package Index). To our knowledge, the only existing Python implementation that offers a reasonably flexible and documented interface is that contained in *dit* [14], a multipurpose information theory toolbox. By focusing narrowly on the IB, embo can offer greater ease of use for the most common applications (by removing the need to preprocess the data and reducing the amount of boilerplate code to a minimum) and support for specialized applications such as the *past-future information bottleneck* [15] (documented more in detail in the notebook located at examples/Markov-Chains.ipynb within the source distribution). Moreover, and very importantly for the application of IB methods to real-world research problems, embo is much more computationally efficient than dit. Figure 3 shows that embo offers a 1000x–10000x speedup over dit on a set of simple problems (embo can solve much larger problems, but these are not included in the comparison because they become prohibitively time-consuming with dit).

Taken together, the features discussed in this section help to remove all barriers in going from empirical data to an IB curve, thus making the IB method more accessible to a broad generalist audience.

### Implementation and architecture

The main point of entry to the package is the InformationBottleneck class. In its constructor, InformationBottleneck takes as arguments an array of observations for *X* and an (equally long) array of observations for *Y*, together with other optional parameters (see the software documentation for details). Alternatively, a joint probability mass function *p*(*x, y*) can be directly specified. In the most basic use case, users can call the get_bottleneck method of an InformationBottleneck object. Embo will then solve the optimization problem in Equation (1) for a certain set of values of *β* and will return the set of solutions, composed of the optimal values of *I*(*M* : *X*), *I*(*M* : *Y*) and *H*(*M*) corresponding to each of those *β*. The IB bound can then be visualised by plotting *I*(*M* : *Y*) vs *I*(*M* : *Y*), as we have done in Figure 1 (top panels). If an alpha argument was passed to the InformationBottleneck constructor, the corresponding GIB problem as per Equation (3) will be solved instead. To visualize the DIB bound, it is then sufficient to specify alpha=0 and plot *I*(*M* : *Y*) vs *H*(*M*), as we have done in Figure 2. Usage examples of InformationBottleneck, illustrating the output to be expected on some sample input data, are given in the software’s documentation.

From the architectural standpoint, embo can parallelize the computation of the IB curve on multicore machines by breaking down the set of *β* values into *k* smaller subsets and running each subset in parallel. This functionality is implemented with the *multiprocessing* Python module and can be controlled by the user by setting an optional parameter specifying the number *k* of processes to use.

Embo has several other optional parameters, which allow the user to control precisely the range and number of *β* values to be considered, as well as finer aspects of the behaviour of the algorithm that solves the optimization problem (3) for a given *β* (the Blahut-Arimoto algorithm [1, 2, 12]) and to automatically preprocess data for the application of the *past-future bottleneck* method [15]. These parameters are all described in the software’s documentation, but embo comes with reasonable defaults allowing users to worry about such details only if needed.

### Quality control

Embo has a suite of unit tests to ensure basic functionality and prevent regressions. These tests are integrated with Gitlab’s continuous integration (CI) pipelines, so that unit tests are automatically run each time new commits are pushed to Gitlab. Tests include running (G)IB analyses on a variety of datasets and probability distributions, both fixed and randomly generated at test time. The tests check properties such as lim_*β*→∞_ *I*(*M* : *Y*) = *I*(*X* : *Y*) and that embo’s internal functions for computing information-theoretic quantities (such as entropy and Kullback-Leibler divergence) give the same results as those provided by SciPy. Tests are automatically run against multiple versions of NumPy using *tox* (https://pypi.org/project/tox/). CI reports are publicly available online at https://gitlab.com/epiasini/embo/pipelines.

Meaningful examples of IB analyses are available as Jupyter notebooks in embo’s documentation. These examples are distributed with the software (for instance when it is installed via *pip*) and are listed in the package’s README and are viewable online at https://gitlab.com/epiasini/embo/-/tree/master/embo/examples. These examples play a double role: as a tutorial on how to use the software, and as a sanity check that the software is behaving as expected. As mentioned in the caption to Figure 1 and explained in much further technical detail in the notebooks, the examples used in the Jupyter notebooks are chosen to make it easy for the user to gauge if embo is behaving correctly. For instance, by construction an IB curve should always lie below the identity line and never include points with coordinates larger than the base 2 logarithm of the number of possible values taken on by the variables being analyzed [1]. These properties can be immediately checked by visual inspection of Figure 1, which is taken from one of the notebooks mentioned above.

The examples available in the documentation also showcase embo’s other features, such as facilities for solving the generalized and deterministic bottleneck problems, parallel computation of GIB bounds and the integrated facility for performing past-future-bottleneck type analyses.

## (2) Availability

### Operating system

Embo is a pure Python package and therefore has ample compatibility. It has been tested to run on Linux (Ubuntu 16.04, 18.04 and 20.04) and macOS (10.13 and 10.14).

### Programming language

Embo requires Python 3.

### Additional system requirements

Embo does not have any special system requirement. It supports parallel computation on multicore machines through the *multiprocessing* module in Python’s standard library.

### Dependencies

Embo requires a recent version of NumPy [16] (≥ 1.17) and SciPy [17]. Matplotlib [18] is recommended to plot IB curves, but is not a dependency. Embo can be installed using *pip*, the de-facto standard Python package management system, by simply running the command pip install embo, but installation from a source code archive (by downloading the source and running python setup.py install) is supported too.

### List of contributors

- Eugenio Piasini, University of Pennsylvania (developer)
- Alexandre Filipowicz, University of Pennsylvania (developer)
- Jonathan Levine, University of Pennsylvania (developer)
- Joshua Gold, University of Pennsylvania (consultant)

### Software location:

Archive (1)

**Name:** Zenodo
**Persistent identifier:** 10.5281/zenodo.3625785
**Licence:** GNU General Public License v3.0 or later
**Publisher:** Eugenio Piasini, Alexandre L. Filipowicz, Jonathan Levine
**Version published:** 1.1.0
**Date published:** 22/02/2021

Archive (2)

**Name:** Python Package Index (PyPI)
**Persistent identifier:** https://pypi.org/project/embo/
**Licence:** GNU General Public License v3.0 or later
**Publisher:** Eugenio Piasini, Alexandre L. Filipowicz, Jonathan Levine
**Version published:** 1.1.0
**Date published:** 22/02/2021

Code repository

**Name:** Gitlab
**Persistent identifier:** https://gitlab.com/epiasini/embo
**Licence:** GNU General Public License v3.0 or later
**Date published:** 22/02/2021

Language

English

## (3) Reuse potential

In [11], Embo has been used to assess the complexity of the strategies adopted by human subjects during cognitive tasks. In the computational cognitive science and neuroscience domain, the same approach can be used to analyze human or animal behavior in different tasks, as well as the statistical relationship between sensory stimuli and recorded neuronal activity [8, 9]. More generally, the Information Bottleneck method is entirely domain agnostic, and embo can be used in any setting involving joint observations of two discrete, low-dimensional variables.

Embo may be extended in several ways. Possible technical upgrades include improving the software’s performance, for instance by rewriting the Blahut-Arimoto algorithm implementation (or some critical paths of it) in C, or by using performance-oriented Python libraries such as Numba or Cython. Features that may be added include the estimation of finite sample bounds for the IB [19]. Finally, embo may be coupled with analyses based on multipartite information decompositions [20, 21] to study the mutual relationship of triplets of empirical variables, where one is hypothesized to act as a bottleneck between the other two. This condition is highly relevant for the study of neural activity recorded concomitantly with sensory stimulation and behavioural output in awake animals [22].

The recommended support channel for Embo is via its GitLab projects, where issues can be reported, and patches and merge requests are welcome. Additionally, the maintainers can be contacted directly at their institutional email addresses.
